# Synergistic Effect of Afatinib with Su11274 in Non-Small Cell Lung Cancer Cells Resistant to Gefitinib or Erlotinib

**DOI:** 10.1371/journal.pone.0059708

**Published:** 2013-03-18

**Authors:** Gang Chen, Alfiah Noor, Peter Kronenberger, Erik Teugels, Ijeoma Adaku Umelo, Jacques De Grève

**Affiliations:** 1 Department of Pathology, First Affiliated Hospital, Guangxi Medical University, Nanning Guangxi, People's Republic of China; 2 Laboratory of Medical and Molecular Oncology, Department of Medical Oncology, Oncology Center, Universitair Ziekenhuis Brussel, Vrije Universiteit Brussel, Brussels, Belgium; 3 Laboratory for Biotechnology, Departement Gezondheidszorg, Erasmushogeschool Brussel, Brussels, Belgium; University of Porto, Portugal

## Abstract

Epidermal growth factor receptor (EGFR) and c-MET receptors are expressed on many non-small cell lung cancer (NSCLC) cells. Current single agent therapeutic targeting of a mutant EGFR has a high efficacy in the clinic, but is not curative. Here, we investigated the combination of targeting EGFR and c-MET pathways in NSCLC cells resistant to receptor tyrosine kinase inhibitors (TKIs), using RNA interference and inhibition by TKIs. Different NSCLC cell lines with various genomic characteristics (H358, H1650 and H1975) were transfected with EGFR-specific-siRNA, T790M-specific-siRNA, c-MET siRNA or the combination. Subsequently EGFR TKIs (gefitinib, erlotinib or afatinib) or monoclonal antibody cetuximab were combined respectively with the c-MET-specific TKI su11274 in NSCLC cell lines. The cell proliferation, viability, caspase−3/7 activity and apoptotic morphology were monitored by spectrophotometry, fluorimetry and fluorescence microscopy. The combined effect of EGFR TKIs, or cetuximab and su11274, was evaluated using a combination index. The results showed that the cell lines that were relatively resistant to EGFR TKIs, especially the H1975 cell line containing the resistance T790M mutation, were found to be more sensitive to EGFR-specific-siRNA. The combination of EGFR siRNA plus c-MET siRNA enhanced cell growth inhibition, apoptosis induction and inhibition of downstream signaling in EGFR TKI resistant H358, H1650 and H1975 cells, despite the absence of activity of the c-MET siRNA alone. EGFR TKIs or cetuximab plus su11274 were also consistently superior to either agent alone. The strongest biological effect was observed when afatinib, an irreversible pan-HER blocker was combined with su11274, which achieved a synergistic effect in the T790M mutant H1975 cells. In a conclusion, our findings offer preclinical proof of principle for combined inhibition as a promising treatment strategy for NSCLC, especially for patients in whom current EGFR-targeted treatments fail due to the presence of the T790M-EGFR-mutation or high c-MET expression.

## Introduction

In some non – small cell lung cancer (NSCLC) patients, the epidermal growth factor receptor (EGFR, also known as ErbB1 or HER1), contains “sensitizing” mutations that increase the efficacy of EGFR-specific tyrosine kinase inhibitors (TKIs) [1], [2]. Two main anti-EGFR strategies are currently in clinical application: low-molecular-weight TKIs that compete with ATP for binding to the tyrosine kinase portion of a mutant EGFR receptor, and monoclonal antibodies (mAbs) that are directed at the ligand-binding extracellular domain, thereby preventing ligand binding, and consequently receptor dimerization, and receptor signaling. These two classes of agents have shown solid preclinical and clinical activity in a variety of tumor types [3].

Among the receptor TKIs, erlotinib (Tarceva, Genentech, Inc, South San Francisco, CA, and OSI Pharmaceuticals Inc., Melville, NY) improves survival in advanced NSCLC patients who progressed after one or two prior chemotherapy regimens [4], [5], [6], [7]. Both gefitinib and erlotinib are superior to chemotherapy in the first-line treatment of lung adenocarcinoma in which the EGFR receptor harbors the sensitizing mutations in exon 19/21 [8], [9], [10]. The aggregated clinical experience today indicates that only patients whose tumors contain a sensitizing mutation, derive a meaningful clinical benefit from EGFR TKIs. In fact, randomized studies indicate that in patients not selected for such mutations, these drugs might have an adverse effect on outcome [10], [11].

The efficacy of the inhibitors is limited in time due to the appearance of cells with resistance mechanisms, in nearly half of the cases a threonine-to-methionine substitution in the EGFR at amino acid position 790 (T790M). Afatinib (BIBW 2992, Boehringer Ingelheim GmbH), is an irreversible inhibitor of both EGFR, HER2 and HER4 kinases and retains some activity in tumors with T790M mutations, but at doses that are a log higher than what is needed for cancers harboring sensitizing mutations [12], [13], [14], [15], [16], [17].

The chimeric IgG1 monoclonal EGFR antibody cetuximab (ERBITUX, ImClone Systems Incorporated, New York, NY, and Bristol-Myers Squibb Company, Princeton, NJ) blocks the ligand-receptor interaction and thereby down-regulates EGFR signaling, resulting in inhibition of cell proliferation and angiogenesis, and induction of apoptosis [3]. Cetuximab in combination with chemotherapy, has been approved by the FDA and EMEA for the treatment of metastatic colorectal cancer (CRC) and in combination with radiotherapy for the treatment of locally advanced head and neck cancer (HNC) [18], [19]. Cetuximab has demonstrated a modest activity as a single agent as well as in combination with docetaxel in patients with advanced, chemotherapy-refractory NSCLC [20]. A multinational, multicentre, open-label, phase III trial has shown that addition of cetuximab to platinum-based chemotherapy improved the outcome for patients with advanced NSCLC [21]. The overall benefit, however, is limited, so that there is no consensus on the relevance for clinical application.

RNA interference (RNAi), by short interfering RNAs (siRNAs) or short hairpin RNAs (shRNAs), has provided a powerful tool with which to modulate gene expression for the study of gene function. RNAi is currently also under consideration as a therapeutic tool, in the laboratory and the clinic [22], [23], [24]. Several reports described effects of EGFR-targeted RNAi to inhibit cell growth [25], [26], [27], [28], [29], however attempts to knock down the T790M-containing allele (using lentiviral shRNA constructs) were unsuccessful [26].

Acquired resistance to TKIs can also develop through a “kinase switch”, with c-MET amplification and over-expression [30], [31]. Amplification of c-MET, a transmembrane tyrosine kinase receptor, can already occur before the treatment with TKIs in NSCLC [32], [33], [34], [35], and c-MET is expressed in 60% of NSCLC tumors as measured by immunohistochemistry [34]. High levels of hepatocyte growth factor (HGF), the c-MET ligand, produced by stromal cells [36], have also been correlated with poor prognosis of NSCLC patients [37]. c-MET amplification and up-regulation were identified in some patients with acquired resistance to gefitinib/erlotinib [30], [31]. In addition, c-MET can be activated by mutations as well [38]. therefore c-MET inhibition could become a valuable treatment strategy for these patients.

In the current study we have first investigated the combined effects of EGFR-specific siRNAs with c-MET siRNA on different NSCLC cell lines with distinct genomic characteristics. We also investigated the combination of EGFR TKIs (gefitinib, erlotinib or afatinib) or the monoclonal EGFR antibody cetuximab, with the c-MET inhibitor su11274 in the same set of lung cancer cell lines.

## Methods

### SiRNAs

Previously, eight siRNAs targeting wild type EGFR and T790M sequences (NCBI Reference Sequence: NM_005228.3) were designed using algorithms from Invitrogen, Eurogentec, Dharmacon, Maurice HO Rational siRNA design (http://ihome.ust.hk/~bokcmho/siRNA/siRNA.html), Reynolds [39], Ui-Tei [40] and Jagla [41]. The capacity of these siRNAs candidates to knock down EGFR or T790M-mutant EGFR at the mRNA level, and their ability to suppress cell proliferation was tested [42]. The most effective siRNAs were selected for the experiments in the current study (Table 1). c-MET siRNAs (NCBI Reference Sequence: NM_001127500.1) were purchased as an “on target smartpool” (Thermo Scientific Dharmacon, Blenheim, England) and the siRNA sequences were summarized in Table 1. Scrambled siRNAs were generated by www.sirnawizard.com and verified by a BLAST search (http://blast.ncbi.nlm.nih.gov/Blast.cgi). The glyceraldehyde-3-phosphate dehydrogenase (GAPDH) positive control siRNA from Invitrogen (ref. SKU#12935-140 Stealth RNAi GAPDH Positive Control, Merelbeke, Belgium) was used as a primary test for the siRNA transfection efficiency. A TOX transfection Control and siGLO Green transfection indicators were performed as positive controls for transfection efficiency (ref. D-001630-01-02; D-001500-01-05, Thermo Scientific Dharmacon, Blenheim, England). The negative control siRNA was a proprietary sequence that does not correspond to any eukaryotic gene (OR-0030-neg 05, Eurogentec S.A., Liege, Belgium). The siRNA duplexes were transiently transfected using lipofectamine^TM^ 2000 (Cat. No. 11668-019, Invitrogen Merelbeke, Belgium) in RPMI-1640 medium containing 10% fetal bovine serum without antibiotics, as described previously [43], [44], [45]. Each experiment was conducted at least in triplicate and three times independently.

**Table 1:** siRNAs used in the study.

Name of siRNA	Sequence	Location and length(nt)	GC content(%)
EGFR siRNA1247	GCAAAGTGTGTAACGGAATAGGTAT	1247–1271(25)	40
Scrambled EGFR siRNA1247	GGTGATTAGGTTATAAACGGACAGA	NA(25)	40
T790M siRNA2600	CCGTGCAGCTCATCATGCAGC	2600–2620(21)	62
Scrambled T790MsiRNA2600	GCGCAGTCGCTAGCTCACTCA	NA(21)	62
Wt for T790MsiRNA2600, EGFRwtsiRNA2600	CCGTGCAGCTCATCACGCAGC	2600–2620(21)	67
c-MET siRNA1188	GAGCCAGCCTGAATGATGA	1188–1206(19)	53
Scrambled c-MET siRNA1188	GGAGCAACGAGGATTACCT	NA(19)	53
c-MET siRNA2950	GAACAGCGAGCTAAATATA	2950–2968(19)	37
Scrambled c-MET siRNA2950	GTGACACGAAACAGTATAA	NA(19)	37
c-MET siRNA3191	GTAAGTGCCCGAAGTGTAA	3191–3209(19)	47
Scrambled c-MET siRNA3191	GTAGCAAGCGACTGATGTA	NA(19)	47
c-MET siRNA4235	GAACTGGTGTCCCGGATAT	4235–4253(19)	53
Scrambled c-MET siRNA4235	GCGATAGCGGTCTGTACTA	NA(19)	53

NA: not applicable.

### Cell lines and reagents

The human NSCLC cell lines H292 (CRL-1848™, mucoepidermoid pulmonary carcinoma), H358 (CRL-5807™, bronchoalveolar carcinoma), HCC827 (CRL-2868™, adenocarcinoma), H1650 (CRL-5883™, adenocarcinoma; bronchoalveolar carcinoma), and H1975 (CRL-5908™, adenocarcinoma) were obtained from the American Type Culture Collection (ATCC, Netherlands). The cell line H292 was reported as an EGFR and K-Ras wild type cell line [46], [47], [48], [49], which we confirmed using real-time RT-qPCR and sequencing analysis (data not shown). H358 is EGFR wild type, has a codon 12 K-Ras mutation [50], and a homozygous deletion of p53 [51]. H1650 and HCC827 have an in-frame deletion mutation in the EGFR tyrosine kinase domain (E746-A750 deletion, exon 19). H1650 cells also have a COOH-terminal deletion of PTEN and loss of PTEN protein [52] and express the insulin-like growth factor receptor (IGF1R) [53]. The cell line H1975 has a sensitizing L858R kinase domain mutation in exon 21, and a second T790M in exon 20, *in cis*, in the EGFR kinase domain, rendering them resistant to the reversible TKIs gefitinib and erlotinib [54]. Moreover, all these five cell lines express the c-MET receptor without gene amplification [28], [30], [53], [55], [56]. All five cell lines were cultured in RPMI 1640 medium (Invitrogen Corp., Gent, Belgium), supplemented with 10% heat-inactivated fetal bovine serum (Perbio Science NV, Erembodegem, Belgium), 2 mM L-glutamine and 1 mM sodium pyruvate at 37°C in a humidified incubator with 5% CO_2_. Ten mM stocks of the EGFR-specific TKIs gefitinib (AstraZeneca, Cheshire, United Kingdom), erlotinib (AstraZeneca, Cheshire, United Kingdom), the irreversible pan-HER inhibitor afatinib (Boehringer Ingelheim, Ingelheim, Germany) and a c-MET inhibitor, su11274 (Sigma-Aldrich N.V. Bornem, Belgium) were prepared in dimethyl sulfoxide (DMSO) and stored at −80°C. These drugs were diluted in fresh RPMI 1640 with a final concentration of DMSO less than 0.1% in all experiments. The EGFR-specific monoclonal antibody cetuximab (2 mg/ml) was purchased from Merck KgaA, Darmstadt, Germany.

### RT-qPCR

RNA isolation, RNA normalization, reverse transcription and qPCR were as described previously [43], [44], [45].

### Western blot analysis

After being treated with siRNAs or different agents for the indicated periods, the cells were washed with PBS and lysed in a buffer containing Tris/HCL (ph 7.6) 20 mM, NaCl 150 mM (ph 6.85), EDTA 1 mM (ph 8), TRITON-X 1%, Na-pyrophosphate 2.5 mM, Sodium orthovanadate (Na3VO4) 1 mM, Leupeptin 1 µg/ml, protease inhibitor cocktails 1% and phosphotase inhibitor cocktails 1% (Sigma-Aldrich NV/SA, Bornem, Belgium). The lysates were centrifuged at 12,000×g for 10 min at 4°C and boiled for 5 min. The protein concentration of the lysate was detected by the Bio-Rad Bradford protein assay (Nazareth Eke, Belgium) and 25 µg of denatured protein was subjected to SDS-PAGE (10% SDS-acrylamide gel) with a loading buffer containing 80 mM Tris–HCl (ph 6.8), 5% SDS,10% glycerol, 5 mM EDTA (ph 8), 5% 2-MercaptoEthanol, 0.2% Bromophenolblue and 1 mM phenylmethylsulfonyl fluoride. The separated proteins were transferred to PVDF membranes (BioRad) for 2 h at 100 mA. The membrane was incubated with the following primary antibodies as indicated: EGFR (Cell Signaling), phospho-EGFR (Tyr1173, clone 9H2, Upstate), c-MET (L41G3, Cell Signaling), phospho-c-MET (Tyr1234/1235, 3D7, Cell Signaling), phospho-AKT/PKB (pS473, Invitrogen), phospho-ERK1/2 (pTpY185/187, Invitrogen), phospho-STAT3 (Tyr705, 3E2, Cell Signaling), phospho-STAT5 (pY694, BD Biosciences) and β-actin (Sigma-Aldrich N.V.). A peroxidase-conjugated secondary antibody (1∶4000 dilution, ECL Anti-mouse or Anti-rabbit IgG HRP linked, Na 931, GE Healthcare Bio-sciences/Amersham, Diegem, Belgium) was added, and the membranes were subjected to chemiluminescence detection (ECL Plus Western Blotting Detection Reagents, GE Healthcare Bio-sciences/Amersham, Diegem, Belgium).

### Proliferation

Cell growth was assessed using a colorimetric tetrazolium assay (substrate MTS, CellTiter96 AQueous One Solution Cell Proliferation Assay G3580, Promega, Madison, USA). The protocol was as follows: different agents and combinations were added to 96 well plates with increasing concentrations, and incubated at 37°C for up to 96 h (combination experiments up to 72 h). Following addition of 20 µl of MTS reagent to each well, the plates were incubated for 2 h at 37°C in a humidified 5% CO_2_ incubator, and the absorbance at 490 nm was recorded using a 96-well microplate reader (Scientific Multiskan MK3, Thermo Finland). The results were the mean of six wells and expressed as the ratio of the absorbance divided by the absorbance of mock control ×100.

### Viability

Cell viability was assayed by fluorimetric detection of resorufin (CellTiter-Blue Cell Viability Assay, G8080, Promega, Madison, USA). The procedure was according to the manufacturer. The treatments and controls were as aforementioned. Fluorimetry (ex: 560 nm/em: 590 nm) was using an FL600 fluorescence plate reader (Bio-Tek, USA). All assays were performed in triplicate and each time six individual wells were used. Fluorescence data are expressed as the fluorescence of treated samples divided by fluorescence of the mock control ×100.

### Caspase−3/7 activity detection

Caspase−3/7 activity was measured using a synthetic rhodamine labeled caspase−3/7 substrate (Apo-ONE® Homogeneous Caspase−3/7 Assay, G7790, Promega, Madison, USA) immediately after the fluorimetric detection of cell viability on the same wells, according to the instructions of the manufacturer. After incubation at room temperature for 60 min, the fluorescence of each well was measured (ex: 499 nm/em: 512 nm), using a FL600 fluorescence plate reader (Bio-Tek, USA). Caspase−3/7 activity is expressed as fluorescence of treated sample/mock control×100.

### Fluorescent microscopy evaluation of cell apoptosis and morphology

The effects of the different treatments on apoptosis and nuclear morphology in the cells were assessed by Hoechst 33342 (Sigma-Aldrich N.V. Bornem, Belgium) and propidium iodide (PI, Sigma-Aldrich N.V. Bornem, Belgium) double fluorescent chromatin staining. In brief, after single or dual treatment of siRNAs or agents, cells were washed with ice-cold PBS and stained 15 min with Hoechst (1 mg/ml) and propidium iodide (PI, mg/ml), and observed under an advanced fluorescence microscope (ZEISS Axiovert 25, Zaventem, Belgium). Apoptosis and nuclear morphology were identified by condensation of nuclear chromatin and its fragmentation. This system determines the absolute number of viable cells (Hoechst positive/PI negative), early apoptotic cells (Hoechst positive/PI negative with blue fragmentations in the cells), late apoptotic cells (Hoechst positive/PI positive, with red fragmentations in the cells), necrotic cells (Hoechst negative/PI positive) and debris signals. Viable and apoptotic cells were counted in 10 different fields under the 200× magnification, and in each well from three independent experiments. Counting was by two persons and the average result was expressed relative to the mock control. Apoptotic cell numbers from different treatments were compared by being normalized to their viable cell numbers.

### Statistical analysis

SPSS19.0 was used for statistical analysis. Results were representative of three independent experiments unless stated otherwise. Values were presented as the mean ± standard deviation (SD). One-way Analysis of Variance (ANOVA) test was used to analyze significance between groups. The Least Significant Difference (LSD) method of multiple comparisons with different treatments and control group was applied when the probability for ANOVA was statistically significant. Statistical significance was determined at a *P*<0.05 level. The analysis of additivity and synergism was assessed by the Biosoft CalcuSyn program (Ferguson, MO, USA). The Combination Index (CI) was used to express synergism (CI <1), additive effect (CI  = 1), or antagonism (CI >1) [57].

## Results

### Effect of EGFR wild type and T790M-specific-siRNAs on target expression and malignant phenotype

**Figure 1:**
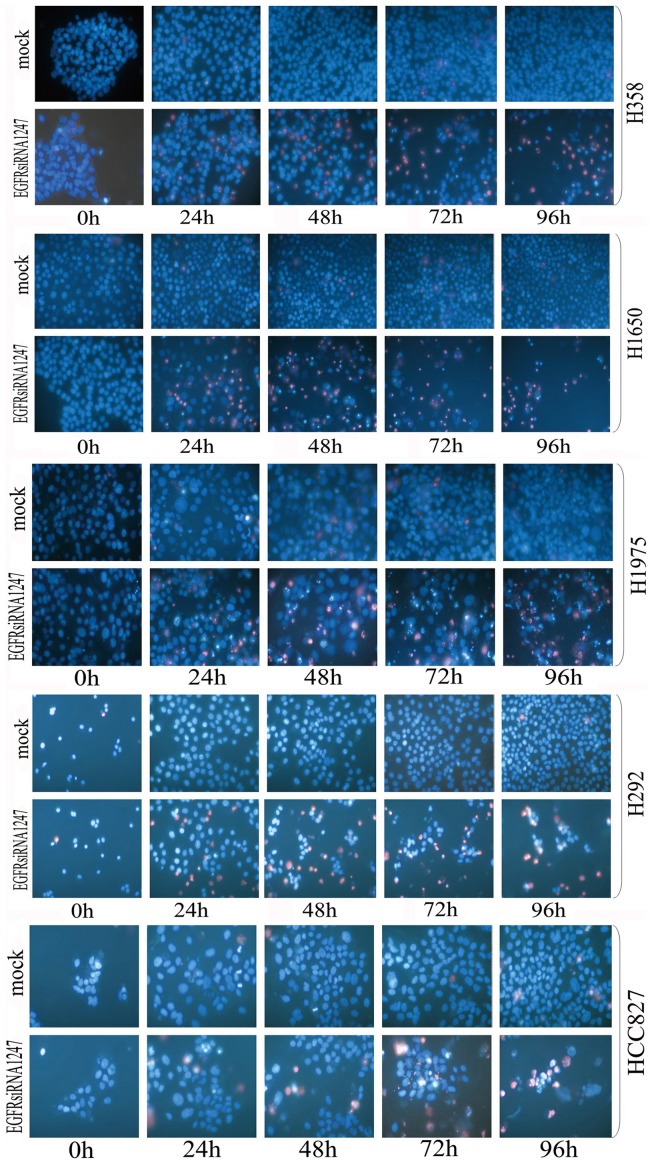
Effects of EGFR-specific-siRNA on cell growth and apoptosis. Lung cancer cell lines were treated with EGFR-specific-siRNA 1247. Live cells and apoptotic cells were detected with Hoechst 33342 and PI double fluorescent staining. The number of apoptotic cells was normalized to the number of live cells in the same well. Hoechst 33342 and PI double fluorescent staining ×200.

The siRNA targeting wild type sequences was able to knock down the EGFR transcript, reduce the EGFR protein level, inhibit cell growth and induce cell apoptosis in all NSCLC cell lines tested, however, with different magnitude independent from the specific driver gene mutations (Figure 1). Likewise, the T790M-specific-siRNA could knock down T790M mRNA, and also suppress cell proliferation and enhance cell caspase activity in H1975 cells containing the T790M mutation. However, the effect was less potent compared to the EGFR-specific-siRNA (data not shown). All negative and scrambled control siRNAs had no effect.

### Dual Targeting of EGFR and c-MET with siRNAs

**Figure 2:**
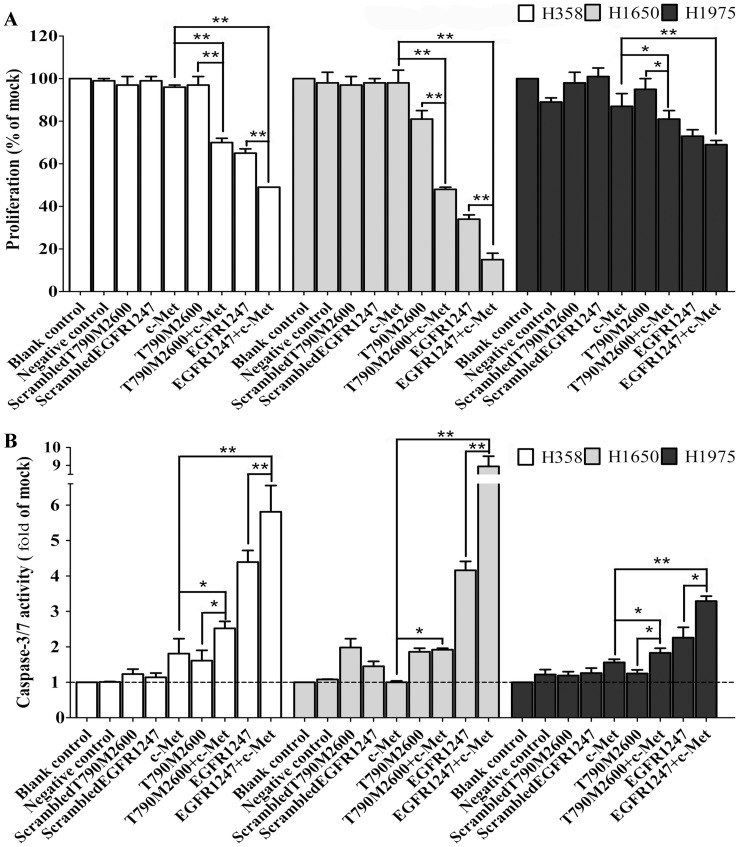
Functional effect of combination of EGFR-specific-siRNA with c-MET siRNAs. **Panel A**: Cell proliferation following transfection with different siRNAs. Cell lines H358 (white), H1650 (grey) and H1975 (black) were transfected with EGFR-specific-siRNA 1247, T790M specific siRNA, a c-MET siRNA pool, or combinations of these, and the proliferation was assayed 72 h post transfection using a colorimetric MTS assay. **Panel B**: Caspase−3/7 activity. * *P*<0.05 and ** *P*<0.01 compared to both single treatment.

**Figure 3:**
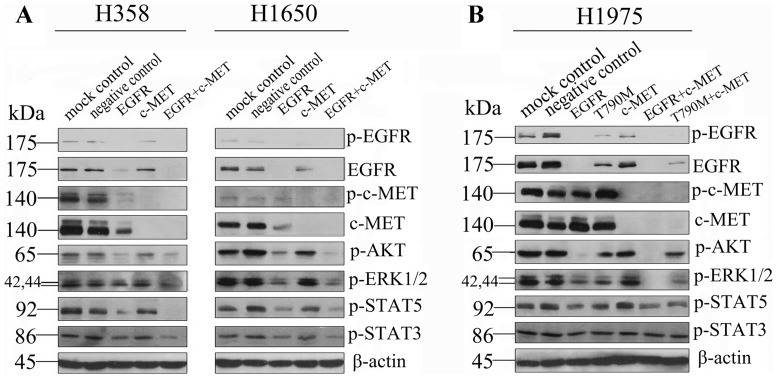
Protein level of combination of EGFR-specific-siRNA with c-MET siRNA. **Panel A**: Immunoblot analysis of H358 and H1650 cells transfected with wild type EGFR siRNA, a c-MET siRNA pool or a combination of these. **Panel B**: Immunoblot analysis of H1975 cells, as in panel C. Here, the T790M-specific-siRNA was also included. Antibodies included phosphorylated (p-) EGFR, EGFR, p-c-MET, c-MET, p-AKT, p-ERK1/2, p-STAT5, p-STAT3, and β-actin.

Cell lines with different mutations (KRAS: H358; PTEN: H1650; T790M: H1975) that render them resistant to EGFR TKIs were further selected for the dual targeting. The resistance of H1975 cells towards EGFR TKIs has been attributed to the presence of the T790M mutation, but also to activation of the c-MET receptor [30], [31]. Synergy of c-MET inhibition with EGFR inhibition has been shown in models that have an activated or overexpressed c-MET pathway [58]. In our experiment we have investigated the interaction of EGFR inhibition and c-MET inhibition in cells where such c-MET activation is not present. A pool of c-MET siRNAs had little or no effect on cell growth (MTS assay) in the cell lines H358 and H1650. In H1975 cells, an extremely modest reduction in the proliferation rate was observed (to 87%, Figure 2A). There was a similar lack of caspase−3/7 induction for the c-MET inhibition only treatment in all cell lines (Figure 2B). Although immunoblotting revealed that the c-MET receptor was effectively knocked down, and that c-MET phosphorylation was inhibited, also in H1975 cells (Figure 3), there was little effect on downstream signaling (AKT, ERK and STAT pathways). c-MET siRNA treatment alone thus has no significant effect on any of the NSCLC cell lines tested *in vitro*.

The addition of EGFR siRNA (whether wild type or T790M-specific)to c-MET siRNAs enhanced growth inhibition in H358 and H1650 cells compared to EGFR siRNA alone, but much less so in H1975 cells (Figure 2A). Combined treatment also increased caspase−3/7 activity. In H1650 cells the apoptosis signal even increased to 8.94-fold of the control compared to single EGFR siRNA (4.16-fold. In H1975 cells, there was a more modest increase to 3.29-fold, which is slightly higher than EGFR siRNA alone (2.26-fold). Western blotting, however, revealed that the combination EGFR/c-MET siRNA was able to affect the ERK pathway as well (in addition to AKT, Figure 3), which is in contrast to EGFR inhibition alone. The ERK pathway was downregulated the most in H1975 cells, whereas the biggest reduction of p-AKT was observed in H1650 cells.

The effect on viability was also assessed using a fluorimetric resorufin viability assay and by microscopic counting of viable (Hoechst 33342 positive/PI negative) cells. In both assays the results mirrored the MTS tetrazolium assay (data not shown). Likewise, caspase−3/7 activity was also confirmed by Hoechst and PI double fluorescent staining assay (data not shown).

### Dual Targeting of EGFR with TKIs or cetuximab and c-MET with su11274

We further verified whether the results obtained with combined siRNA treatment could be mimicked using dual EGFR and c-MET-specific inhibitors that can be more easily applied *in vivo*. Lung cancer cells were treated with the single EGFR TKIs gefitinib, erlotinib, or afatinib, and in combination with c-MET inhibitor su11274. The combination of the monoclonal EGFR antibody cetuximab with su11274 was also studied.

**Figure 4:**
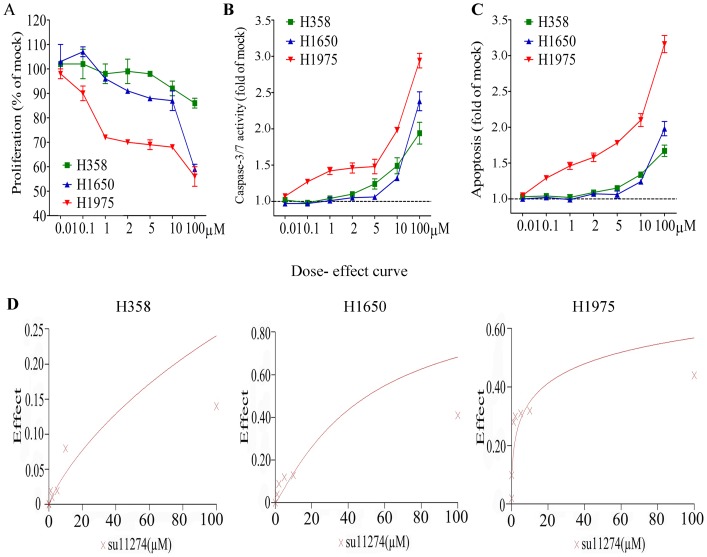
Effect of su11274 in lung cancer cells containing TKI resistance mutations. H358, H1650 and H1975 cells were incubated with a concentration range of su11274, and the cells were incubated for 72 h. **Panel A:** proliferation. **Panel B:** caspase−3/7 activity. **Panel C:** apoptosis. **Panel D:** dose-effect curves.

**Figure 5:**
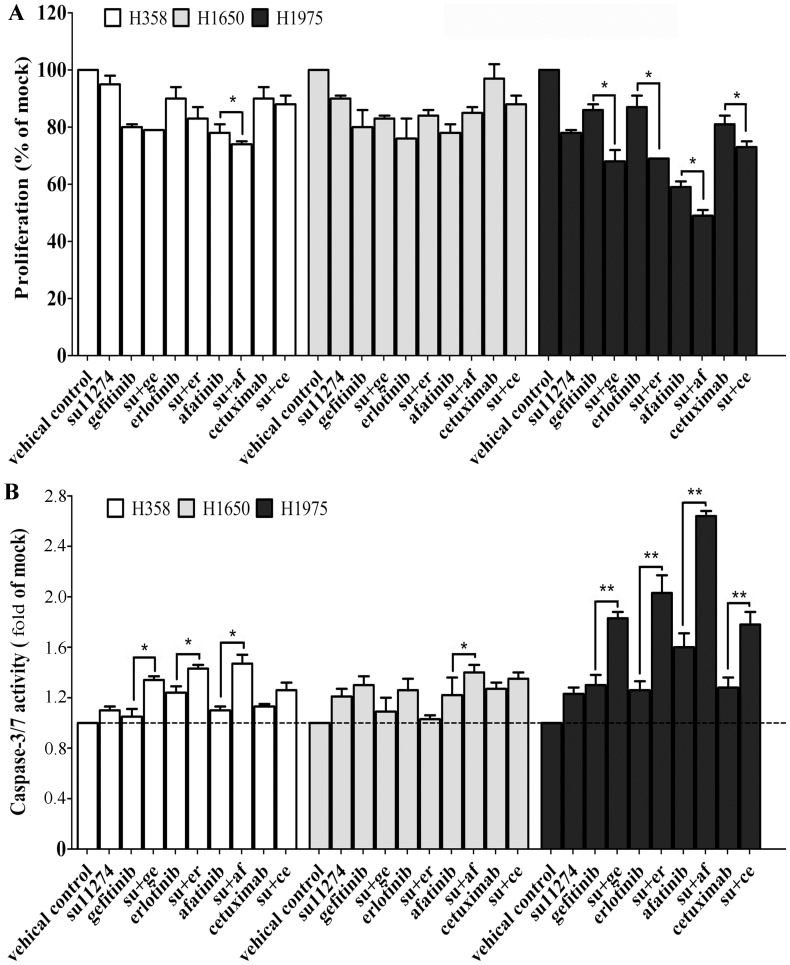
Combination of EGFR-specific TKIs or cetuximab and the c-MET inhibitor su11274. **Panel A**: proliferation of cells following single or combined TKI treatment. Cell lines H358 (white), H1650 (grey) and H1975 (black) were cultured in RPMI containing 10% FBS and 1 µM su11274, gefitinib, erlotinib, afatinib or cetuximab, and their combinations. The proliferation was assayed 72 h post treatment using a colorimetric MTS assay. **Panel B**: caspase −3/7 induction by single or combined TKI treatment. * *P*<0.05 and ** *P*<0.01 compared to both single treatment.

The effect of su11274 alone on cell growth and apoptosis was extremely weak in all three cell lines, although, there was a slightly stronger effect in H1975 cells, which has higher c-MET expression than the other two cell lines (Figure 4). At a concentration of 1 µM su11274, for example, a reduction of 20–30% in the proliferation rate and a modest increase of 40–50% in caspase−3/7 signals contrasted to a lack of effect in the two other cell lines (Figure 4, Figure 5). These results are congruent with the c-MET siRNA experiments.

In the combination with EGFR TKIs, the concentrations were maintained at 1 µM for all the agents. In H358 and H1650 cells (Figure 5A), the TKIs alone induced a small reduction in cell growth (maximally by 41% in H1975 with afatinib, Figure 5A), and a modest increase of caspase−3/7 signals (by 1.6-fold-at maximum also in H1975 with afatinib, Figure 5B) compared to the vehicle controls. The combinations with su11274 were able to decrease cell growth slightly further in H358 and H1650, and the combination effect was more potent in H1975 (notably su11274+ afatinib, Figure 5A). Meanwhile, the addition of su11274 offered some gain in caspase−3/7 signals, in accordance with the cell growth inhibition data (Figure 5B). The strongest effects were seen in the H1975 cells, known to be EGFR-TKI resistant. In these cells, single-agent treatment with gefitinib and erlotinib is ineffective, while afatinib can reduce growth and induce apoptosis. All the combinations with su11274 achieved an enhanced effect on cell growth and apoptosis. The combination su11274+ afatinib was able to reduce cell viability to 49% at 1 µM concentration (Figure 5A), and increased apoptotic signals to 2.64-fold (Figure 5B).

**Figure 6:**
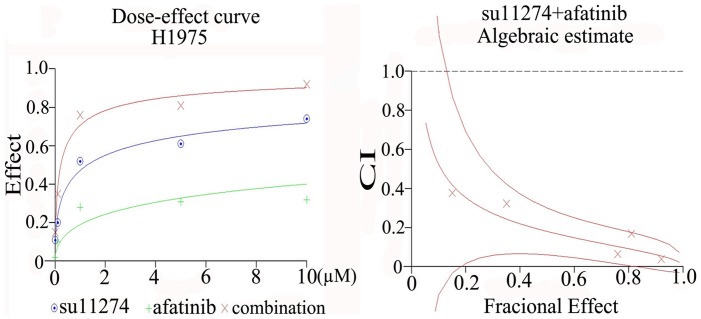
Combination Index of EGFR-specific TKIs or cetuximab and the c-MET inhibitor su11274. combination index (CI) was calculated in H1975. CIs >1, indicating additive effect (**Panel A:** gefinib+su11274; **Panel B:** erlotinib+su11274; **Panel C:** cetuximab+su11274).

**Figure 7:**
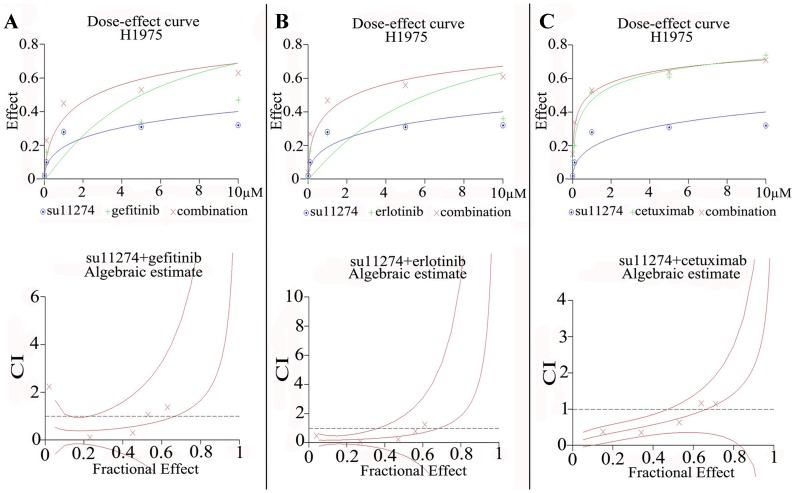
Combination effect of afatinib and su11274 in H1975 cells. Combination Index (CI) of afatinib and su11274. Here, CI of afatinib + su11274<1, indicating synergistic effect.

**Figure 8:**
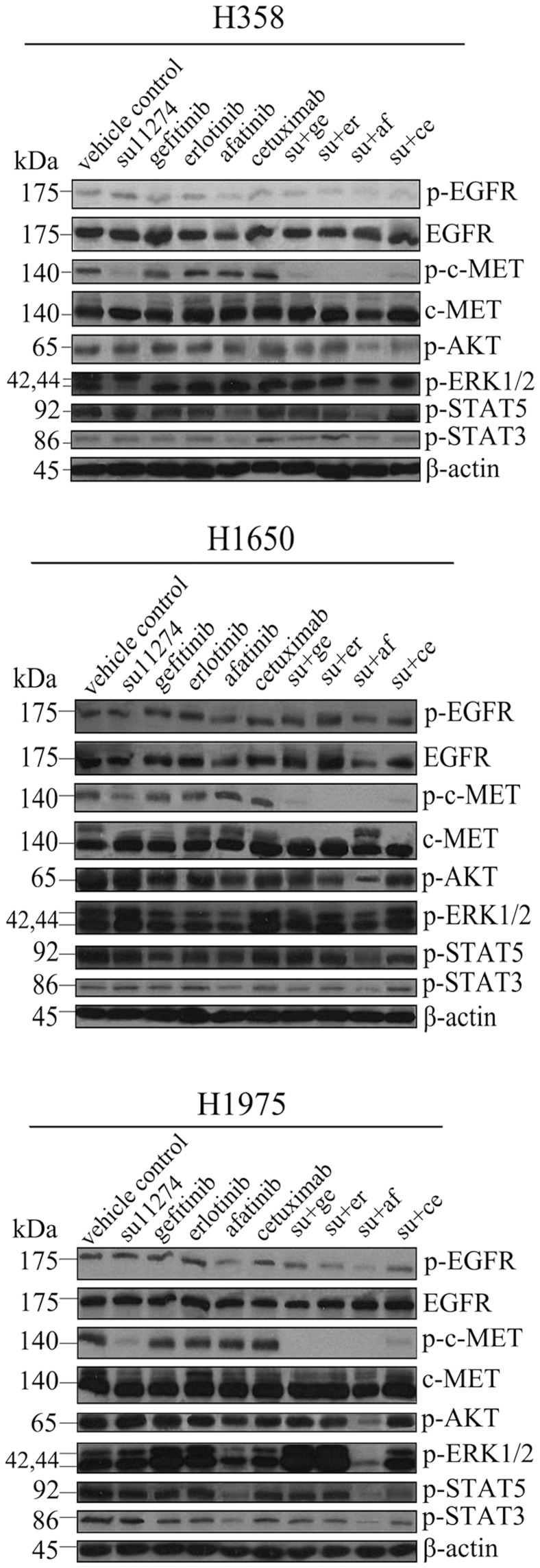
Protein level of combination of EGFR TKIs or cetuximab and the c-MET inhibitor su11274. Immuno blotting of cell lysates of H358, H1650 and H1975 cells treated with single or combined TKIs/cetuximab. Antibodies included phosphorylated (p-) EGFR, EGFR, p-c-MET, c-MET, p-AKT, p-ERK1/2, p-STAT5, p-STAT3, and β-actin.

In order to ascertain the additive or synergistic nature of the dual treatments, a series of concentrations up to 1 µM was set up for each compound and the combinations. The effects were scored using the colorimetric MTS formazan proliferation assay, and a CI that differentiates between additive and synergistic effects was calculated (Biosoft Calcusyn software). The results unambiguously show that the combined effect of all the agents on proliferation in H358 and H1650 was additive. In H1975 cells, an additive effect from gefitinib or erlotinib or cetuximab + su11274 was also observed (Figure 6). However, the combination of afatinib + su11274 scored synergistically (CI <1) in cell growth inhibition and apoptosis induction (Figure 7). This synergistic effect could be explained by cooperative inhibition of the proliferative/survival and anti-apoptotic signal pathways AKT, ERK and STAT (Figure 8). Western blot suggested that inhibition of these three pathways is particularly effective in H1975 cells following combined treatment with su11274+ afatinib (Figure 8).

## Discussion

EGFR is a therapeutic target in NSCLC. In a previous study we have shown that single agent drug targeting with EGFR TKI does not achieve a maximal biological effect, even in sensitive cell lines, and that the addition of EGFR-specific-siRNA to EGFR TKIs or cetuximab increases the therapeutic effects [45]. However, only targeting the EGFR pathway is not effective in cells that harbor resistance mechanisms and is even insufficient to annul the full malignant phenotype in sensitive cells. Thus the next question was whether simultaneous inhibition of EGFR and c-MET, by RNAi, would result in increased biological effects, and therefore be useful for overcoming TKI resistance in H1975 cells. Tang et al [28] showed that the combination of EGFR siRNA and c-MET siRNA in H1975 cells resulted in enhanced inhibition of downstream EGFR signaling, including the pro-survival AKT and STAT3 pathways. Here, we explored the effect on cell growth and apoptosis. The addition of c-MET siRNA to either EGFR wild type or T790M-specific siRNAs, resulted in an increased effect on cell growth inhibition and caspase−3/7 activity induction. The EGFR wild type siRNA was more potent than T790M siRNA, in agreement with single siRNA results described above, and consistent with the degree of pathway inhibition (see below). The combination with c-MET siRNA increased growth inhibition and apoptosis induction more significantly in H358 and H1650 cells. Immunoblotting shows a consistent down-regulation of phospho-AKT, consistent with Tang et al. [28], but the down-regulation of phospho-STAT3 was weaker. We also found that two downstream signals were inhibited: phospho-STAT5 and especially phospho-ERK1/2. When T790M and c-MET siRNAs were combined, the cooperative effect was less potent.

The dual targeting of EGFR and c-MET was then verified using TKIs (gefitinib, erlotinib and afatinib plus su11274). Single agent treatment with either inhibitor, in the three EGFR TKIs resistant cell lines had a relatively weak effect on growth inhibition and apoptosis induction and was even minimal or absent for the c-MET inhibitor. However, higher doses of erlotinib could induce apoptosis in the KRAS mutant cell line H358 [45]. In the present study, we found that a combination of su11274 and erlotinib also had a higher effect on cell growth inhibition in H1975 cells. The degree of inhibition (by 35%) was in the same range as reported by others (by 42%) [28]. To ascertain the additive or synergistic nature of the dual treatment, a series of increasing TKI concentrations was set up. The effects were evaluated using the colorimetric MTS formazan proliferation assay, and a CI was calculated. The effect of dual erlotinib plus su11274 treatment in H1975 cells was additive. A similar additive effect was found with gefitinib or cetuximab plus su11274 in H1975 cell. However, the combination of afatinib and su11274 clearly had a synergistic effect on cell growth inhibition in H1975 cells. This was also reflected in the cooperative inhibition of phospho-AKT, phospho-ERK1/2, phospho-STAT3 and phospho-STAT5 (Figure 8). A synergistic effect was not observed with the other TKI combinations, suggesting that the synergy arises from the pan-HER blockage by afatinib and/or the fact that it binds EGFR more potently.

The effects of EGFR and c-MET combination on cell proliferation in H358 and H1650 have not been reported previously with any TKI. In the present study, we showed that the combined effect of all the agents on proliferation in H358 and H1650 was additive.

We next studied the effect on apoptosis of the combination of EGFR TKIs and su11274. We showed that gefitinib combined with su11274 caused significantly higher caspase−3/7 activity than either of the single treatment in H358 cells. This is in agreement with Puri, et al. [59], showing that gefitinib and su11274 induced apoptosis in NSCLC cell line H358 in a synergistic fashion, as determined by FACS analysis. In the same cell line H358, the combination of erlotinib or afatinib with su11274 also induced higher caspase−3/7 activity. But in H1650 cells, only the combination of afatinib and su11274 had a higher induction of apoptosis.

The by far most pronounced biological effect was observed in H1975 cells when afatinib was combined with su11274. In contrast to what could be observed in the other cell lines or with the other TKIs for which smaller and additive effects were observed, this treatment yielded a synergistic inhibition of growth and induction of apoptosis. This combination has not been reported before and these data thus provide a strong rationale for the *in vivo* and clinical exploration of this combination specifically in patients whose tumors carry a T790M- resistance mutation with or without activated c-MET.

Interestingly, there were important differences between the effect of RNAi and pharmacological inhibition. First, H358 and H1650 cells were much more sensitive to simultaneous inhibition by RNAi of both EGFR and c-MET pathways, but less effect was observed when pharmacological inhibitors were used. This could be explained by the different mechanisms of these two approaches: the oncogenic activity of EGFR and c-MET emanates from the activation of signaling that promotes both cell proliferation and cell survival. When the mRNAs are knocked down, ERK kinases contributing substantially to the proliferative activity and AKT and STATs largely linking to anti-apoptosis are also affected, as evidenced by the western blots. Conversely, inhibition by kinase inhibitors depends on the sensitivity of a particular EGFR (mutations) towards inhibition, and resulted in a variable and less powerful responses. In the H358 and H1650 cell lines, the influence on the downstream pathways by pharmacological inhibition was indeed weaker compared to RNAi, as observed in immuno blotting. A second difference is that in H1975 cells, the power of RNAi versus the TKI combination is exactly the opposite. TKI-resistant H1975 cells were indeed more sensitive to combined pharmacological inhibition than by RNAi. This result can be explained by the more potent pan-HER blockage by afatinib in this cell line, which is noted to block not only EGFR signaling but also, the other HER family members (Boehringer Ingelheim, data on file, [17]). Most recently, Tabara et al [60] have reported observations that might point in the same direction as the present study. In the erlotinib-resistant cells from PC9 established by Tabara et al [60], constitutive PI3K/Akt activation was effectively inhibited by afatinib. Moreover, HER2 overexpression and amplification were reported recently to be a potential mechanism of acquired resistance to EGFR inhibition in EGFR mutant lung cancers that lack the second-site EGFR T790M mutation [61]. This would suggest then, that HER2 and HER4 signaling have become important for survival in resistant H1975 cells with T790M mutation, but less so in H358 and H1650 cells. This hypothesis has been confirmed by RNAi combining EGFR, HER2 and HER4 in H1975 cells, which causes 90% cell growth suppression (data to be reported elsewhere).

## Conclusions

In summary, the results obtained in the present study indicate that despite the absence of a c-MET amplification in H358, H1650 and H1975 cells, the c-MET signal pathway is functional and that inhibition of c-MET can enhance the effects of EGFR inhibition, in spite of c-MET inhibition alone having no effect. This was paralleled in experiments using TKIs against both targets. The by far most impressive activity was seen when afatinib and a c-MET small molecule inhibitor su11274 are combined, leading to synergistic inhibition of growth and induction of apoptosis. Our results implicate that this combined targeting (afatinib plus su11274) may represent a novel strategy to overcome resistance to EGFR TKIs (late use) or prevent such a resistance from emerging (early use). A combined strategy in a first line setting might be preferable to prevent the outgrowth of T790M subclones. However, selecting such patients clinically in a first line setting might not be easy, and might require using deep sequencing methods to detect a minor T790M clone. Further *in vivo* experiments are warranted to assess the therapeutic effect of this combined targeting.
